# Ergot—Its Active Principles

**Published:** 1876-04

**Authors:** 


Ergot and its Active Principles.—Dr. G. Levi has a long and instructive article in *Lo Sperimentale*, for August and September, 1875, in which he gives the results of his study of the physiological and therapeutical action of secale cornutum, especially in its application to obstetrics. He has endeavored to discover how its constituents act in individual cases. From all the facts collected, and from the investigations he has made, the following conclusions are derived :

1. The sum of the therapeutical effects derived from ergot are due to the presence in it of phosphoric acid.

2. The diseases for which ergot is a useful remedy receive equal benefit from phosphoric acid.

3. With ergot two distinct series of phenomena are reproduced by elements of different natures : the physiological (ergotism) recognize as a cause the vegetable principles (ergotine, erboline, etc.) ; the obstetrical, on the contrary, the phosphoric acid.

4. The hæmostatic effects, like the erbolic, are obtained equally, and with the same intensity and promptness, with phosphoric acid as with secale cornutum.

5. The quantity of soluble phosphoric acid found by analysis in recently pulverized ergot, both in that which is old and in the hydro-alcoholic extract (ergotina), is proportional to the obstetric effects which are obtained from these various substances used separately.

